# Craving Responses to Methamphetamine and Sexual Visual Cues in Individuals With Methamphetamine Use Disorder After Long-Term Drug Rehabilitation

**DOI:** 10.3389/fpsyt.2018.00145

**Published:** 2018-04-19

**Authors:** Shucai Huang, Zhixue Zhang, Yuanyuan Dai, Changcun Zhang, Cheng Yang, Lidan Fan, Jun Liu, Wei Hao, Hongxian Chen

**Affiliations:** ^1^Department of Psychiatry, The Fourth People's Hospital of Wuhu, Wuhu, China; ^2^Department of Medical Imaging, The Second Xiangya Hospital, Central South University, Changsha, China; ^3^Pingtang Isolated Compulsory Drug Rehabilitation Center in Hunan Province, Changsha, China; ^4^Department of Psychiatry, The Second Xiangya Hospital, Central South University, Changsha, China; ^5^Hunan Key Laboratory of Psychiatry and Mental Health, Chinese National Clinical Research Center on Mental Disorders (Xiangya), Chinese National Technology Institute on Mental Disorders, Mental Health Institute of the Second Xiangya Hospital, Central South University, Changsha, China

**Keywords:** methamphetamine, long-term drug rehabilitation, cue-reactivity, fMRI, medial prefrontal cortex

## Abstract

Studies utilizing functional magnetic resonance imaging (fMRI) cue-reactivity paradigms have demonstrated that short-term abstinent or current methamphetamine (MA) users have increased brain activity in the ventral striatum, caudate nucleus and medial frontal cortex, when exposed to MA-related visual cues. However, patterns of brain activity following cue-reactivity in subjects with long-term MA abstinence, especially long-term compulsory drug rehabilitation, have not been well studied. To enrich knowledge in this field, functional brain imaging was conducted during a cue-reactivity paradigm task in 28 individuals with MA use disorder following long-term compulsory drug rehabilitation, and 27 healthy control subjects. The results showed that, when compared with controls, individuals with MA use disorder displayed elevated activity in the bilateral medial prefrontal cortex (mPFC) and right lateral posterior cingulate cortex in response to MA-related images. Additionally, the anterior cingulate region of mPFC activation during the MA-related cue-reactivity paradigm was positively correlated with craving alterations and previous frequency of drug use. No significant differences in brain activity in response to pornographic images were found between the two groups. Compared to MA cues, individuals with MA use disorder had increased activation in the occipital lobe when exposed to pornographic cues. In conclusion, the present study indicates that, even after long-term drug rehabilitation, individuals with MA use disorder have unique brain activity when exposed to MA-related cues. Additionally, our results illustrate that the libido brain response might be restored, and that sexual demand might be more robust than drug demand, in individuals with MA use disorder following long-term drug rehabilitation.

## Introduction

Methamphetamine (MA) is an amphetamine-type stimulant that is often used as a recreational drug. It enters the central nervous system (CNS) quickly, resulting in the rapid onset of euphoria, which further motivates drug abuse [1]. In 2014, over 35 million individuals abused amphetamine and methamphetamine worldwide. MA abuse has become a serious public health problem for countries around the world, particularly in China [2]. Since 2016, the number of synthetic drug (MA mainly) users has dramatically increased to 1.51 million, accounting for 60.5% of all registered illicit drug users in China. Furthermore, approximately 360,000 first-time synthetic drug users were recorded in 2016, accounting for 81% of all first-time illicit drug users that year [3].

Drug addiction is a chronic disease characterized by a high rate of relapse [4]. In China, for example, the relapse rate for heroin users, within 2 years of abstinence, is over 90% [5, 6]. Limited data from two follow-up studies demonstrated that the relapse rate in individuals abusing MA, within 1 year following drug rehabilitation, was at least 50% [7, 8]. Drug cravings are very important in the etiology of psychostimulant use relapse [9]. It has been reported that MA cravings decrease within 2 months of abstinence [10]; however, detailed characteristics of MA cravings, including the relationship between the duration of rehabilitation and craving levels, in MA use disorder remain understudied [11].

In recent years, the use of functional magnetic resonance imaging (fMRI) cue-reactivity paradigms has greatly expanded our understanding of the neurobiology underlying addiction and relapse, by providing an opportunity to test the mechanisms by which interventions influence behavior [12]. Recent studies utilizing these techniques have shown that patients with MA use disorder have elevated drug cravings and increased brain activation in interconnected limbic regions (i.e., ventral striatum, cingulate cortex, caudate nucleus, orbitofrontal cortex and medial prefrontal cortex (mPFC) when exposed to drug-related visual cues [13, 14]. However, the patterns of brain activation, following the presentation of MA-related cues, are still poorly understood in long-term abstinent MA abusers.

Reward-related behaviors can be divided into two categories: (1) natural rewards caused by eating, sexual opportunity, and other behaviors in favor of survival and reproduction; (2) drug-related rewards caused by the powerful motivational effects of psychoactive substances on natural reward circuits, which are not necessary for survival and reproduction. It is well accepted that pleasure caused by addictive drug use tends to be more rapid, more robust, and longer lasting than natural rewards. It is also known that the euphoric effects of different addictive drugs are diverse, depending on their respective pharmacological mechanisms. Results from an fMRI study suggested that, when compared with healthy controls, both current heroin users and ex-heroin users were less responsive to sexual cues, but had increased activation in the prefrontal and temporal cortex when exposed to drug cues [15]. Unlike opiates, MA use probably does not lead to robust pleasure, as some people use MA to improve their own sexual performance [16, 17]. As a result of different biological mechanisms, the cortical response to drug and sexual cues in individuals with MA use disorder might be different from those of opiate users. However, empirical evidence is still lacking.

In this study, we attempted to explore the patterns of cortical activation in the brains of individuals with long-term MA abstinence when exposed to drug- and sexual-related visual cues using fMRI cue-reactivity paradigms. In light of the high relapse rate in MA use disorder, we hypothesized that (1) cravings in individuals with MA use disorder would still be intense following at least 16 months of drug rehabilitation, and (2) drug-related cues might arouse cravings accompanied by specific brain region activation.

## Materials and methods

### Subjects

This was a case-control study. Participants included 28 long-term abstinent MA addicts and 27 age-matched healthy volunteers.

Participants with long-term MA abstinence were recruited from the Pingtang Isolated Compulsory Drug Rehabilitation Center in Hunan Province. For the convenience of presenting uniform pornographic images in the fMRI cue-reactivity paradigm, only males were included in the study. Further inclusion criteria were: aged between 18 and 45 years; of Han Chinese ethnicity; right-handedness; meeting Diagnostic and Statistical Manual of Mental Disorders (DSM-IV) criteria for MA dependence, as determined by interviews conducted using the Chinese version of the Structured Clinical Interview for DSM-IV axis I disorders, research version for patients (SCID-I/P) [18]; a history of MA use > twice per week and for >2 years; a duration of drug abstinence >16 months. Exclusion criteria were: illiteracy; a lifetime diagnosis of substance dependence other than MA and nicotine; current or past major medical, neurological or axis I psychiatric disorders; current use of psychotropic medications or intravenous drugs; learning disabilities or CNS disease; a history of head injury with skull fracture or loss of consciousness of 10 min or more; homosexuality; and contraindications for MRI.

Healthy controls were local residents, who were male, 18–45 years old, of Han Chinese ethnicity, and right-handed. Those who were current or past MA users, met DSM-IV criteria for any axis I, major medical, and/or neurological disorders were excluded.

All subjects were required to abstain from alcohol and/or other potential psychoactive substances for at least 48 h prior to scanning. Meanwhile, all healthy volunteers were required to abstain from all sexual behavior for at least 3 days prior to scanning.

All study procedures were conducted in accordance with the ethical standards of the 1975 Helsinki Declaration. The ethical review board of the Second Xiangya Hospital of Central South University approved all study procedures. All participants were fully informed about research procedures and signed informed consent.

### Measures

Urine screening was conducted to detect the current use of MA, ketamine, opiates, and cocaine prior to the interview and MRI scan. All clinical interviews were conducted by an experienced psychiatrist. The self-rated questionnaires and SCID-I/P were used to collect demographic and drug use information, and to make diagnoses of psychiatric disorders, respectively. The Edinburgh Handedness Inventory [19] was employed to determine the handedness of all participants. MA craving scores and sexual behavior were assessed on a 0–10 visual analog scale (VAS) [20] (0 for the weakest craving and 10 for the strongest craving) prior to and immediately following each MRI scan.

### Procedure

#### Task design

**Figure 1:**
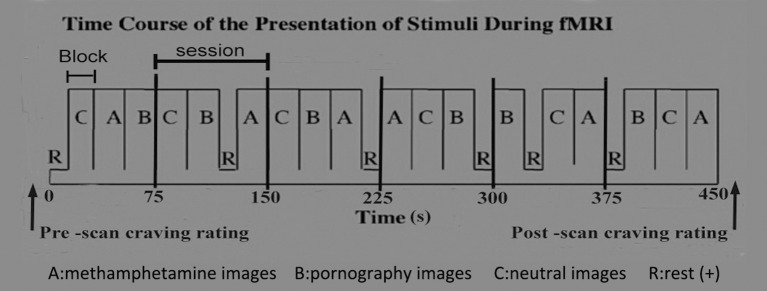
Procedure of fMRI cue-reactivity paradigms in this study.

The cue-reactivity paradigm was utilized to carry out this study. The cue-reactivity paradigm has been widely used to assess the involvement of neurobiological pathways in the processes underlying cravings for alcohol, nicotine, cocaine, and opioids [12]. The cue-induced MRI scanning procedure was designed based on previous reports by George et al. [21], Myrick et al. [22], and Myrick [23], with minor modifications. Briefly, as illustrated in Figure 1, a 450-s sequence for cue-image presentation, consisting of six epochs, was designed. The duration of each epoch was 75 s and contained three 20-s blocks and a 15-s rest. The 20-s blocks presented MA-related, pornographic and neutral images, respectively. The 15-s rest displayed a crosshair. Each 20-s block contained five different images, each displayed for 4 s. A total of 30 MA-related images, 30 sex-related images, and 30 neutral images were presented during the MRI scan, and each image was unique. In order to control for time and order effects across subjects, the order of the images, the blocks within the epoch, and the epochs were all randomly presented [21–23].

All MA-related images, which fall into MA samples, drug paraphernalia and simulation scenarios, were authentic and shot using an SLR camera by researchers. MA samples were provided by the pharmacology laboratory of the Hunan Public Security Bureau. Drug paraphernalia and simulation scenarios of drug use were self-developed and modified after testing by individuals with MA use disorder from the Xinkaipu Isolated Compulsory Drug Rehabilitation Center. Pornographic images were Asian related and high definition (HD). Neutral images were acquired from the internet, including images of artifacts and neutral daily actions. All images were scaled to the same size, resolution and hue through the use of Photoshop (Adobe, San Jose, California) software.

The reliability of all images in the present study were tested preliminarily, and results showed that all images had satisfactory reliability. Each MA-related image was scored (0–10) by 156 individuals with MA use disorder from the Xinkaipu Isolated Compulsory Drug Rehabilitation Center, according to their subjective feelings. We then selected 30 of the 150 images in accordance with the scores. Finally, these 30 images were screened by another 100 individuals with MA use disorder, and the results showed that the recognition rate of these images was 100%. Pornographic and neutral images were checked in the same way as described above. The validity of these images was not tested due to a lack of reference images.

The task paradigm was created and presented in E-prime 2.0 software (http://www.pstnet.com/eprime.cfm) on a computer which was connected to MRI-compatible nonferro-magnetic goggles. Pupil trajectory of all participants was recorded using a mini camera located in the goggles. Participants were required to press a button, which was connected to a computer, when they saw the images clearly. Reaction time (RT), i.e., time taken to press the button, was recorded. A RT longer than 2 s for each image was disqualified and this data was removed from the analysis. The action of planning and pressing is related to executive function, which affects other functional activity in the brain. Therefore, functional image data at the corresponding time points of button pressing were excluded from the analyses.

#### Functional MRI data acquisition

Scanning was conducted in a 3.0 Tesla Siemens scanner (Allegra; Siemens Medical System, Erlangen, Germany) equipped with a standard head volume coil. For fMRI scanning, whole brain echo-planar images were acquired using a T2 weighted gradient echo sequence with blood oxygen level-dependent (BOLD) contrast: repetition time (TR) = 2,000 ms, echo time (TE) = 30 ms, flip = 80°, field of view (FOV) = 220 mm × 220 mm, voxel size = 3.4 mm × 3.4 mm × 4.0 mm, slice thickness = 4 mm, gap = 1 mm, matrix = fMRI scanning 64 × 64, number of slice = 36. The total time of the fMRI scan was 450 s. Earplugs, and cushions placed around the head were used for sound insulation and to control head movement, respectively.

### Statistical analyses

#### Demographics and behavioral data analysis

Comparisons of demographic and behavioral data between MA users and healthy controls were performed with either independent-sample *t*-tests (continuous variables) or χ^2^ tests (categorical variables). Comparisons of increased craving scores (ICS) before and after scanning were performed using paired-sample *t*-tests. SPSS 18.0 software (SPSS, Chicago, Illinois) was used for all analyses. The level of statistical significance was set at *P* < 0.05 (two-sided).

#### fMRI data analysis

Functional images were transferred into an appropriate format for analysis with the Statistical Parametric Mapping 8 software package (SPM 8, http://www.fil.ion.ucl.ac.uk/spm/software/spm8/). First, all functional images were realigned to the first volume of each session as a reference. After this realignment, data sets were selected on the basis of their quality (scan stability), as demonstrated by small motion correction. The realigned images were then stereotactically normalized into a standard resolution of 3 × 3 × 3 mm voxels using the Montreal Neurological Institute (MNI) EPI template in SPM 8, and subsequently smoothed with a 6-mm full-width at half-maximum (FWHM) Gaussian kernel. In the first level of statistical analysis, predetermined conditions effecting entire functional brain volume were analyzed using a boxcar function, convolved with the modeled hemodynamic response function, as the general linear model. Contrast maps were obtained which reflected the differences between MA vs. neutral control, sex vs. neutral control, MA vs. rest, sex vs. rest, and MA vs. sex for each individual. The resulting first level contrast images were entered into second level (random effects) analyses for between-group comparisons. We included age, education, nicotine and RT as covariates in the second level model. To assess the effects of stress, one-sample *t*-tests were performed in all subjects and in each group. Imaging results were corrected using a family-wise error rate for comparisons (significance at *p* < 0.05) [24, 25].

As we had no priori hypotheses for the activity of brain regions, one-sample *t*-test contrasts between MA vs. neutral control (and sex vs. neutral control) were performed for each group, using whole brain analysis with a statistical threshold of *P*_FWEcor_ < 0.05 and *k* = 30 voxels. Two-sample *t*-tests were adopted to detect the main differences between the MA group and the control group.

The voxel locations of significant MA or pornographic cues activated in two groups (*P*_FWEcor_ < 0.05 and *k* = 30 voxels) were used to create masks for time course extraction, and 6-mm radius spherical regions of interest (ROI) were used to create masks. Using the masks, averaged time courses of multi voxels were calculated from each individual data with MarsBar 0.44 (http://marsbar.sourceforge.net/) and log-roi-batch v2.0 (http://www.aimfeld.ch/) [24].

#### Correlation analysis between MA-related variables and activation level of ROIs

Correlations between activation level of ROI and clinical features of MA abuse, including years of age and education, age of starting MA use, duration (months) of MA use, dosage (g) of MA per time, frequency of MA use, craving score when using MA, smoking status, craving score before/after scanning and ICS, were calculated by Pearson correlation. Two-tailed levels of significance (*P* < 0.05) were used.

## Results

### Demographic and behavioral data

There were no significant differences in age and education status between the two groups. Detailed information on demographics and past drug use characteristics of individuals with MA use disorder and healthy controls are displayed in Table 1.

**Table 1:** Demographics and drug use characteristics of subjects with MA use disorder and control subjects.

	**Subjects with MA use disorder**	**Healthy control subjects**
**Cases**	**28**	**27**
**DEMOGRAPHICS**		
Age (years)	31.68 ± 7.06	33.93 ± 7.21
Education (years)	8.96 ± 2.03	10.04 ± 3.03
Male (%)	28 (100%)	27 (100%)
Right-handed (%)	28 (100%)	27 (100%)
**MA USE VARIABLES**		
Age of first use	25.18 ± 7.14	–
Range (years)	15–40	–
Duration of drug use (months)	59.96 ± 32.98	–
Range (months)	24–190	–
Previous frequency of drug use (days per year)	222.71 ± 114.41	–
Range (days per year)	72–365	–
Duration of abstinence (months)	18.50 ± 2.64	–
Range (months)	16–24	–
**OTHER DRUGS EVER USED**		
Alcohol (%)	13 (46.42%)	12 (44.44%)
Cigarette^a^	28 (100%)	17 (62.96%)
Ketamine^b^	14 (50%)	–
Ecstasy^b^	3 (10.71%)	–
Marijuana^b^	1 (3.57%)	–

*The results were presented as mean ± SD*.

a *Significantly different from control group, P ≤ 0.01*.

b *Recreational use, the frequency of drug use was < 25 times during the participants' lifetime*.

Subjective drug craving scores in the MA group were significantly higher after, than prior to, scanning [1.16 (*SD* = 1.27) vs. 0.39 (*SD* = 0.62), *t* = 5.03, df = 27, *P* < 0.01]. In contrast, drug craving scores in the control group were 0 at all times of the study.

Subjective sex craving scores in the MA group were significantly higher after, than prior to, scanning [5.43 (*SD* = 1.94) vs. 2.51 (*SD* = 1.43), *t* = 11.40, df = 27, *P* < 0.01]. Additionally, sex craving scores in the control group were significantly higher after, than prior to, scanning [4.67 (*SD* = 1.71) vs. 2.25 (*SD* = 1.22), *t* = 9.43, df = 26, *P* < 0.01]. Nevertheless, no significant differences were found between the two groups in subjective sex craving scores (before: *t* = 0.72, df = 53, *P* = 0.47, after: *t* = 1.54, df = 53, *P* = 0.13, ICS: *t* = 1.37, df = 53, *P* = 0.18).

After scanning, subjective craving scores for sex were significantly higher than craving scores for drugs in the MA group (*t* = 9.77, df = 54, *P* < 0.01).

### fMRI data

**Figure 2:**
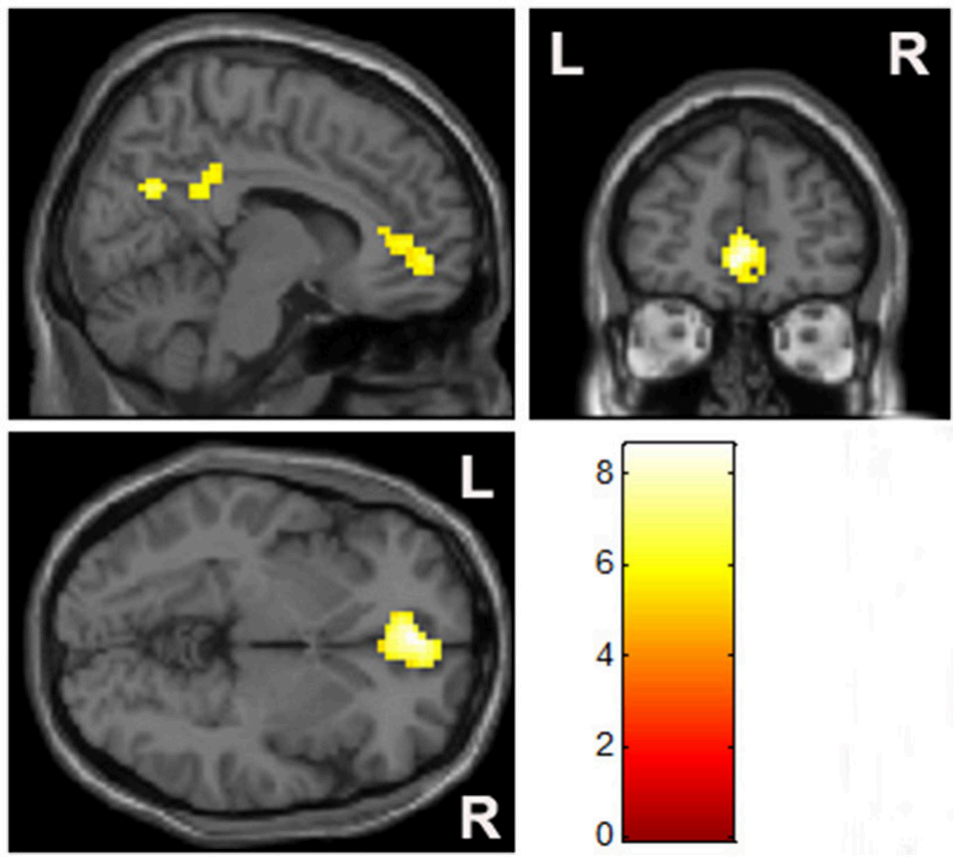
Group comparisons of brain activation when exposed to MA-related images (*p* < 0.05, family-wise error rate corrected).

Compared with the control group, there was a significantly elevated response to MA-related cues in the mPFC and right lateral posterior cingulate cortex, relative to the baseline control condition in the MA group (Figure 2, Table 2).

**Table 2:** Brain regions significantly activated by MA-related images in individuals with MA use disorder when compared with healthy controls.

**Region**	**Hemisphere**	**Montreal neurological institute coordinate**			**Voxels**	***t***	**p_(FWE correction)_**
		***X***	***Y***	***Z***			
Medial prefrontal cortex	Inter	0	48	−3	267	8.60	<0.001
Posterior cingulate cortex	Right	9	−45	27	36	5.95	0.006

**Figure 3:**
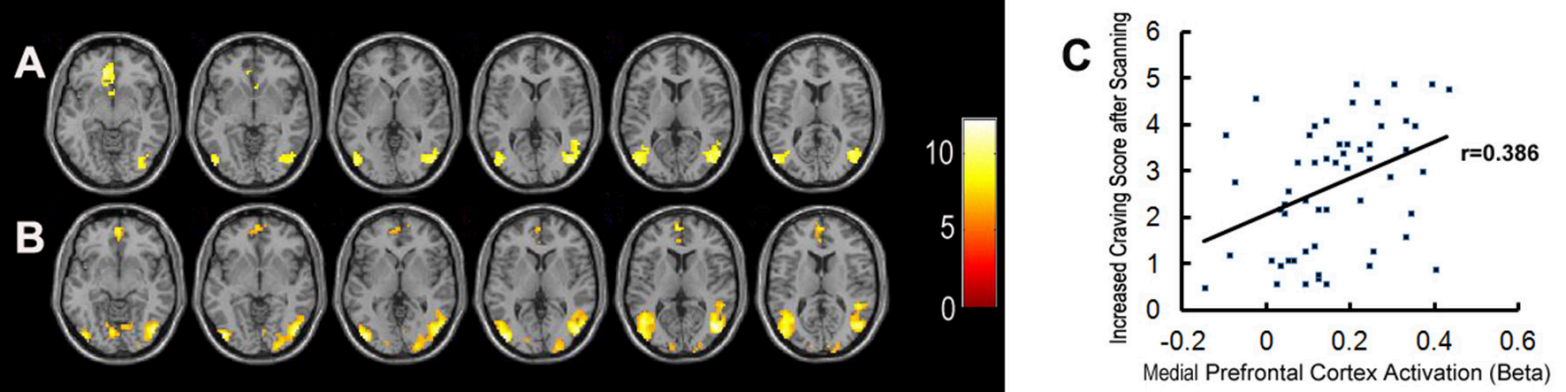
Brain activation when exposed to pornographic images. **(A)** Shows significant activation in brain regions in the healthy control group (*n* = 27). **(B)** Shows significant activation in brain regions in the MA group (*n* = 28). **(C)** Shows the correlation between activation in the medial prefrontal cortex and craving scores for sex in all participants.

Bilateral mPFC, and the occipital and temporal gyrus were activated significantly in both the MA and control groups, when exposed to pornographic images (Figure 3). However, no significant differences in brain activity, in response to sex-related cues, were detected between the two groups.

**Figure 4:**
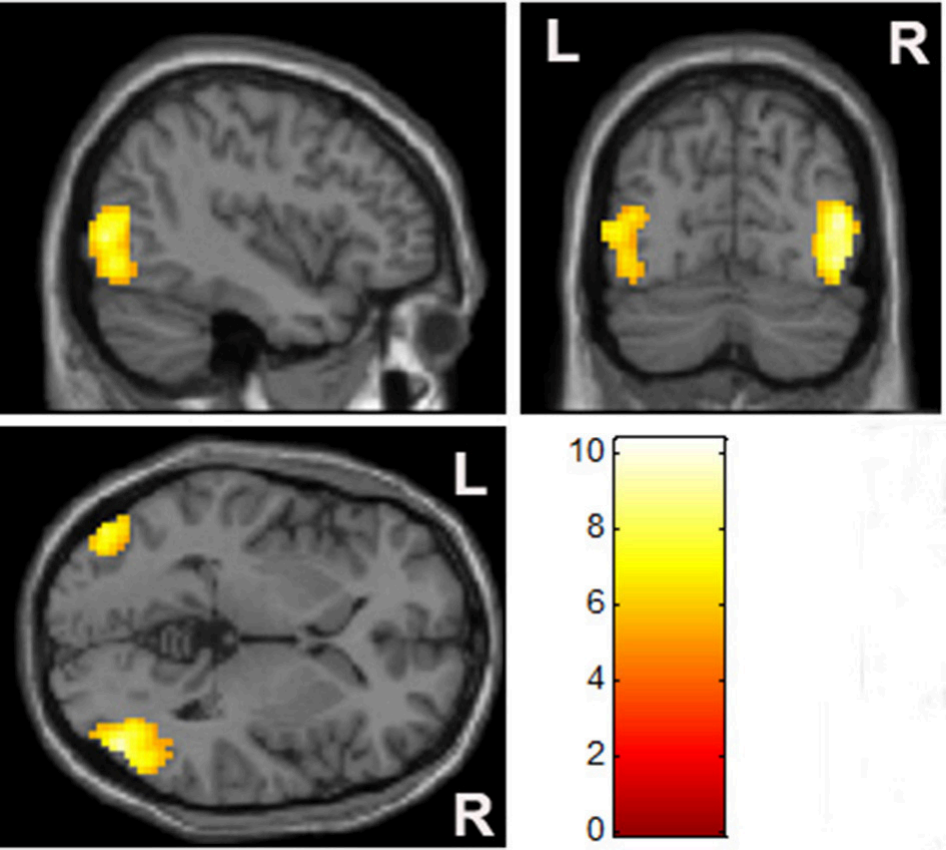
Comparison of brain activation between pornographic images and MA-related images. The bilateral occipital cortex was significantly activated by pornographic images when compared with MA-related images in the MA group (*p* < 0.05, family-wise error rate corrected).

When compared to MA-related image cues, individuals with MA use disorder had elevated left lateral occipital and bilateral temporal gyrus responses, but no reduction in brain activity, when exposed to pornographic image cues (Figure 4, Table 3).

**Table 3:** Brain regions significantly activated by pornographic images when compared with MA-related images in the MA group.

**Region**	**Hemisphere**	**Montreal neurological institute coordinate**			**Voxels**	***t***	**p_(FWE correction)_**
		***X***	***Y***	***Z***			
Inferior occipital gyrus	Right	45	−78	−6	479	7.65	<0.001
Middle occipital gyrus	Left	−42	−81	3	243	6.77	<0.001

### Correlation between demographic or behavioral variables and the activation level of ROIs

**Figure 5:**
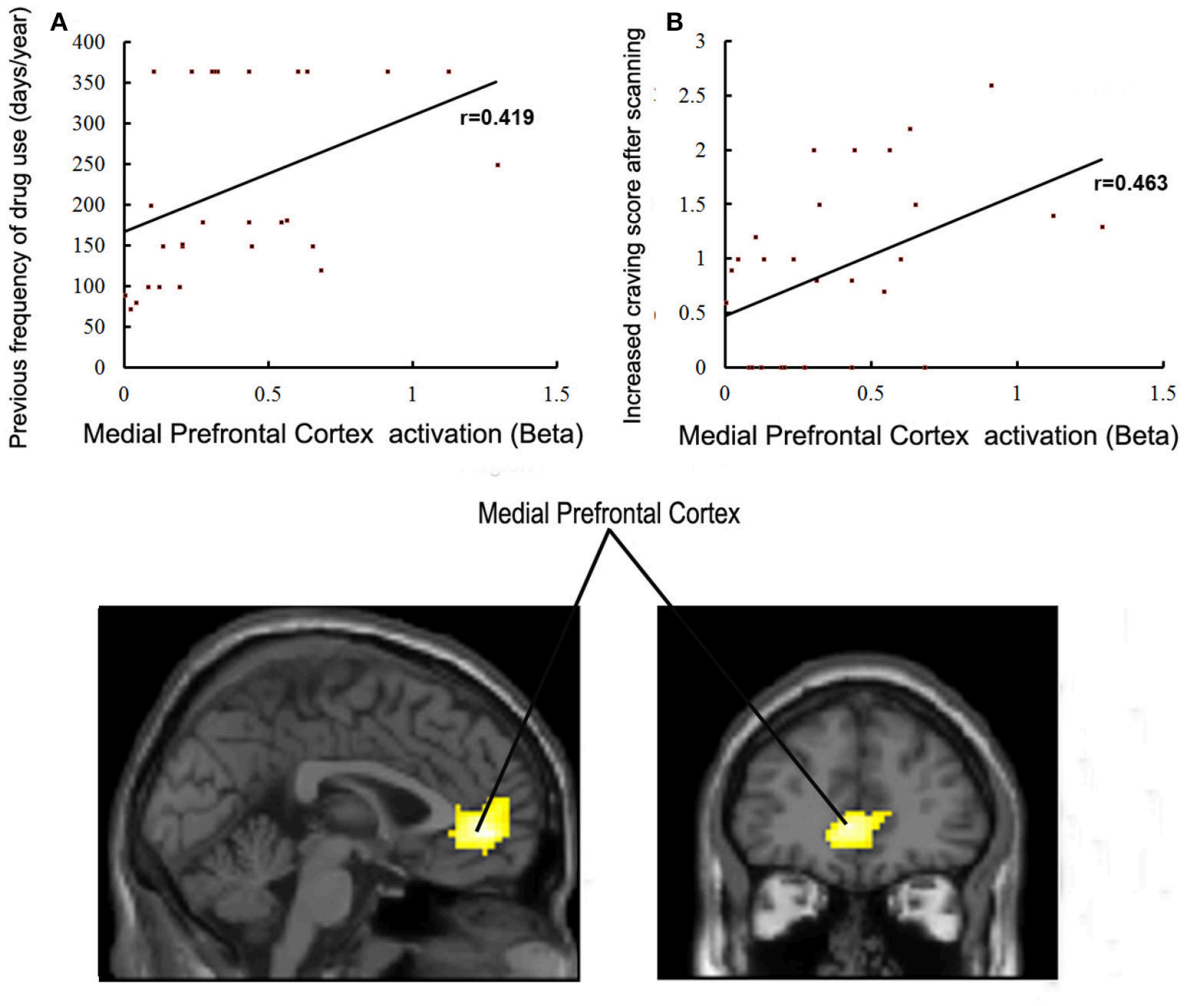
**(A)** Correlations between activation in the medial prefrontal cortex and previous frequency of drug use in MA group (*p* < 0.05). **(B)** Correlations between activation in the medial prefrontal cortex and craving scores for drugs in the MA group (*p* < 0.05).

Brain regions displaying significant activation to MA-related image cues in MA groups (including bilateral mPFC, occipital and temporal gyrus) were selected as the functional ROIs. Correlations between the activation level of these selected ROIs and years of age, years of education, age of starting MA use, duration (months) of MA use, dosage (g) of MA use per time, previous frequency of drug use, craving score when MA using, craving score for MA before/after scanning, and ICS in patients with MA use disorder were examined. The activation level of the left lateral anterior cingulate region of the mPFC was positively correlated with previous frequency of MA use (*r* = 0.419, *P* = 0.027) and ICS for MA (*r* = 0.463, *P* = 0.013) (Figure 5). We did not find any other significant correlations between variables of MA use and activation levels of ROIs.

Meanwhile, brain regions which were significantly activated in response to sex-related images in both groups (including bilateral mPFC, occipital and temporal gyrus) were selected as functional ROIs. The correlations between the activation level of these ROIs and craving scores sex before/after scanning and ICS in all participants were examined. The activation level in a part of the mPFC was positively correlated with ICS for sex (*r* = 0.385, *P* < 0.01) (Figure 3). We did not find any other significant correlations between variables of subjective craving for sex and activation levels of selected ROIs.

## Discussion

Studies of fMRI cue-reactivity paradigms in nicotine [26], alcohol [22], cocaine [27], and MA [14] dependence have identified two interacting circuits that are involved in the process of cue-induced craving: (1) a reward circuit including the nucleus accumbens, ventral tegmental area, amygdala, thalamus, hippocampus and mPFC cortex; (2) a visual attention and planning circuit including the occipital cortex, parietal cortex, and the frontal cortex [28]. Consistent with previous studies, we also found that participants with long-term MA abstinence had significantly increased brain activation in the mPFC (including ACC) and posterior cingulate gyrus when exposed to MA cues, compared to healthy controls. This indicates that MA-related cues may trigger cravings through the reward circuit, and get more attention via the planning circuit in individuals with MA use disorder after long-term drug abstinence. These findings support the theory that ordinary long-term abstinence may not completely improve the extraordinary brain response to MA-related cues, in individuals with MA use disorder.

Previous studies have shown that the mPFC is involved in drug cravings, compulsive seeking behavior and relapse, and that individuals with dysfunction in the ACC of the mPFC display reduced impulse control and enhanced drug-seeking behaviors [29–31]. In our present study, we further found that the activation in the ACC region of the mPFC, when exposed to drug-related cues, was positively correlated with the previous frequency of MA use and ICS in individuals with MA use disorder. This suggests that the responsivity in the ACC region of the mPFC to drug cues could partly reflect a previous history of MA abuse and a current degree of cravings for individuals with MA use disorder. Accordingly, we speculate that the drug-cue-induced activation in the ACC region of the mPFC is a potential predictor of previous MA abuse and current craving level. Additionally, we found that few patients with MA use disorder had aspiration to get rid of drug abuse (7/28). This might be one of the important reasons explaining how the significant increase in the ACC region of the mPFC activity remained in these individuals after long-term abstinence. This is consistent with the results of a previous investigation [27] which demonstrated that drug-cue-induced activation in the middle frontal gyrus was negatively correlated with the level of willingness and motivation to abstain from drug. Therefore, we propose that the lack of motivation to quit for these individuals might be an important risk factor in relapse, and that intensive psychotherapies focusing on drug rehabilitation motivation enhancement and relapse prevention are necessary in the process of drug rehabilitation.

In this work, we also found that pornographic cues produced no significant differences, except for a notable increase in the activity in some areas of occipital lobe, when compared with the activation following MA-related cues in the MA group. It has been reported in previous studies that similar cortical areas in the occipital lobe involved in the processes of visual information, and sensory representation of visual stimulation is processed in these lobes according to the previous experiences, leading to instinctive attention biases to specific visual representation [32, 33]. Therefore, our results might suggest that the brains of individuals with MA use disorder are more interested in sexual visual stimulation, rather than drug-related visual cues, with long-term drug abstinence, which are supported faintly by significantly higher subjective craving scores for sex than drugs in the MA group. This speculation needs to be verified by more rigorous studies.

It has been reported that MA use can improve sexual performance as a result of increased pleasure and extended sexual intercourse, resulting from a transient increase in the release of monoamine neurotransmitters and androgens [16, 17]. However, there is evidence that chronic MA exposure reduces sexual motivation in a dose-dependent manner in humans and experimental animals due to chronic neurotoxicity, monoamine neurotransmitter attenuation, and regulation disorder of androgens, and that these effects of MA probably do not last following drug abstinence due to the recovery of androgens and monoamine neurotransmitters to some extent [34–36]. Consistent with these studies, the final finding of our study is that no significant differences existed in individuals with MA use disorder after long-term drug abstinence in both subjective craving scores and brain responses to pornographic cues, when compared with healthy controls. This demonstrates that the libido of the participants may have been restored. Conversely, a significant reduction in activation of the prefrontal cortex of individuals with heroin use disorder, when exposed to pornographic cues, has been reported in one previous study [15]. These differences of brain activation in response to sexual cues in individuals with different drug use disorders are very interesting.

Several limitations need to be noted when interpreting the results of this study. First, our work is a cross-sectional comparison only, and we could not collect MRI data from these individuals prior to abstinence. Therefore, we could not compare the brain response to related cues before and after abstinence. In addition, due to cultural traditions and stigma of drug abuse, it was difficult to recruit individuals with MA use disorder after long-term voluntary drug abstinence. Therefore, it is not possible for us to compare the differences in cue-induced brain activity between compulsory and voluntary abstinence. Third, the individuals with MA use disorder that we recruited were all from compulsory drug rehabilitation centers. These participants might report a lower level of drug cravings for some reasons; this might introduce bias to the results of the correlation analyses.

In summary, our study reveals patterns of brain activity following exposure to different image cues (neutral, drug, and sex) in healthy controls and individuals with MA use disorder after long-term drug rehabilitation. Extremely enhanced activation remained in the mPFC and posterior cingulate cortex following drug-related cues in individuals who underwent long-term drug rehabilitation. Meanwhile, our results also suggest that the drug-cue-induced activation in the ACC region of the mPFC is positively correlated with a previous history of MA use and the current degree of cravings in individuals with long-term drug abstinence. Additionally, the present study found no significant difference in both subjective craving scores and brain responses to pornographic cues in patients with long-term MA abstinence, when compared with healthy controls. However, a remarkable increase in the activation of some areas involved in the processing of visual information in the occipital lobe, induced by pornographic cues, was found when comparing the brain activation following MA-related cues. This indicates that the brain response to libido might be restored, and the sexual demand might be more robust than drug demand, in individuals with MA use disorder after long-term drug abstinence.

## Author contributions

SH, HC, and WH conceptualized and designed the research; ZZ, JL, and CY performed the experiments; SH and LF undertook the statistical analysis; SH and WH wrote the first draft of the manuscript; HC and WH contributed to the final manuscript. All authors critically reviewed content and approved final version for publication.

### Conflict of interest statement

The authors declare that the research was conducted in the absence of any commercial or financial relationships that could be construed as a potential conflict of interest.
